# Long-Distance Dispersal after the Last Glacial Maximum (LGM) Led to the Disjunctive Distribution of *Pedicularis kansuensis* (Orobanchaceae) between the Qinghai-Tibetan Plateau and Tianshan Region

**DOI:** 10.1371/journal.pone.0165700

**Published:** 2016-11-02

**Authors:** Wen-Jun Li, Xiao-Lin Sui, Patrick Kuss, Yan-Yan Liu, Ai-Rong Li, Kai-Yun Guan

**Affiliations:** 1 Key Laboratory of Biogeography and Bioresource in Arid Land, Xinjiang Institute of Ecology and Geography, Chinese Academy of Sciences, Urumqi 830011, China; 2 University of Chinese Academy of Sciences, Beijing 100049, China; 3 Yunnan Key Laboratory for Research and Development of Wild Plant Resources, Kunming Institute of Botany, Chinese Academy of Sciences, Kunming 650201, China; 4 Institute of Systematic and Evolutionary Botany, University of Zurich, 8008 Zurich, Switzerland; Institute of Botany, CHINA

## Abstract

Quaternary climate fluctuations have profoundly affected the current distribution patterns and genetic structures of many plant and animal species in the Qinghai-Tibetan Plateau (QTP) and adjacent mountain ranges, e.g. Tianshan (TSR), Altay, etc. In this greater area disjunct distributions are prominent but have nevertheless received little attention with respect to the historical processes involved. Here, we focus on *Pedicularis kansuensis* to test whether the current QTP and TSR disjunction is the result of a recent Holocene range expansion involving dispersal across arid land bridge(s) or a Pleistocene range fragmentation involving persistence in refugia. Two chloroplast DNA spacers were sequenced for 319 individuals from 34 populations covering the entire distribution range of this species in China. We found a total of 17 haplotypes of which all occurred in the QTP, and only five in the TSR. Overall genetic diversity was high (*H*_T_ = 0.882, *H*_S_ = 0.559) and higher in the QTP than in the TSR. Genetic differentiation among regions and populations was relatively low (*G*_ST_ = 0.366) and little evidence for a phylogeographic pattern emerged. The divergence times for the four main lineages could be dated to the early Pleistocene. Surprisingly, the two ubiquitous haplotypes diverged just before or around the Last Glacial Maximum (LGM) and were found in different phylogenetic lineages. The Species Distribution Model suggested a disappearance of *P*. *kansuensis* from the TSR during the LGM in contrast to a relatively constant potential distribution in the QTP. We conclude that *P*. *kansuensis* colonized the TSR after the LGM. The improbable long-distance dispersal by wind or water across arid land seed flow may well have had birds or men as vector.

## Introduction

Tectonic events and climate fluctuations have profoundly shaped the current distribution patterns and genetic structures of many plant and animal species in temperate zones of the Northern Hemisphere [1–4]. Since the early Cenozoic, the geology and topography of East Asia underwent dramatic changes. Most notably is the uplift of the Qinghai-Tibetan Plateau (QTP) and adjacent mountain ranges, e.g. Tianshan (TSR) and Altay Mts., which entailed pronounced climatic and environmental dynamics in both space and time [5–7] and a strong effect on landscape and vegetation [8,9]. One consequence is the intense aridification of the Tarim Basin in northwestern China [10–13], resulting in an arid area of about 6.00×10^5^ km^2^ between the QTP and the TSR [14].

The present day distribution of plant and animal species is strongly influenced by these historical processes which potentially and iteratively led to range shifts, range expansion, range contraction and/or range fragmentation. In this context, a disjunct distribution could either be the result of long-distance dispersal from a source area into a suitable new area [15–18] or the consequence of disruption of the previously continuous distribution range [19,20]. Phylogenetic relationship [16–18] and genetic diversity within a given species [21–24] are two aspects frequently considered in order to unravel the historical processes involved. Theoretical and empirical evidence suggests that, when the disjunction is due to recent long-distance dispersal, individuals from separated regions will cluster together in a phylogenetic tree [16–18]. Additionally, the regions are characterized by different levels of genetic diversity [23,25] with the newly colonized region harboring lower levels. By contrast, in the case of range fragmentation, individuals from different regions will cluster by region [18,23] while levels of genetic diversity remain comparable [19,20,24]. Obviously, the level and spatial distribution of genetic diversity within a species is also dependent on the combination of life-history traits, e.g. longevity, breeding system [26,27], which can mask the genetic imprint of historical processes.

Although numerous phylogeographical studies have been carried out in either the QTP [4,28,29] or the greater Tianshan-Altay region [19,21,30–34], investigations addressing the historical processes that led to disjunct distributions are scarce. The limited data available show that plant species had low genetic diversity in Tianshan-Altay region, indicating a rapid colonization from the QTP and strong founder effects in the Tianshan-Altay region [35–37]. However, these studies included either samples from only one Altay population, i.e. the congeneric *Pedicularis longiflora* [35], or plant species less representative of highland terrestrial plants, i.e. the fern *Lepisorus clathratus* and the aquatic *Hippuris vulgaris* [36,37]. Thus, it remains questionable whether the current QTP and TSR disjunctions are the result of a recent Holocene range expansion involving dispersal across arid land bridge(s) or a Pleistocene range fragmentation involving persistence in refugia.

Here, we focus on *Pedicularis kansuensis* Maxim. (Orobanchaceae), a highland plant species widespread in western China and Nepal, with a disjunct distribution between the QTP and the TSR but not known from the Altay. This species was previously mis-identified as *P*. *verticillata* in the TSR [38–41], but clarified to be *P*. *kansuensis* based on morphological and molecular evidence [42]. It is an annual or facultative biennial hemiparasitic herb, occurring in moist gravelly ground or grassy slopes in subalpine zone at elevations between 1,800 and 4,600 m [43]. In nearly twenty years, *P*. *kansuensis* has been reported to rapidly expand in population sizes and become weedy in Bayanbulak Grassland of the Tianshan Mts., which has caused great loss of herbage yield and threatened the local livestock industry [39,44].

In the present study we aim to unravel the historical processes that led to the current disjunctive distribution of *P*. *kansuensis*. Given the great extent of today's arid Tarim Basin which separate the QTP and the TSR we propose two alternative scenarios: (a) *P*. *kansuensis* survived the LGM *in situ* or in refugia in the respective foothills. Here we would expect a strong phylogeographic signal, existence of unique regional haplotypes and comparable high levels of genetic diversity. (b) *P*. *kansuensis* colonized the TSR from the northern fringes of the QTP via long-distance seed dispersal after the LGM. Under this scenario we would expect to find a certain degree of genetic similarity between the source and sink regions, i.e. shared haplotypes, but not a strong phylogeographic signal. Also, the sink region would be characterized by lower levels of genetic diversity and evidence for rapid population expansion should be detectable.

## Materials and Methods

### Ethics statement

This study was conducted in accordance with the laws of the People’s Republic of China. No specific permits were required for accessing the sampling locations. *P*. *kansuensis* is not an endangered or protected species.

### Plant sampling

Leaf tissue of *P*. *kansuensis* was collected from 34 populations across the Qinghai-Tibetan Plateau (QTP) and the Tianshan region (TSR) in western China (Table 1). Three to 16 individuals growing at least 20 m apart were sampled in each georeferenced population rendering a total of 319 individuals. Fresh leaves were dried in silica gel and stored at room temperature until DNA extraction. For all populations voucher specimens were deposited at the Herbarium of the Xinjiang Institute of Ecology and Geography, Chinese Academy of Sciences, Xinjiang, China (XJBI)(S1 Table).

**Table 1:** Details of sample locations, samples size (N), haplotypes, haplotype diversity (h) and nucleotide diversity (π) of 34 populations of *Pedicularis kansuensis* surveyed for DNA sequence variation at two combined chloroplast regions.

Population code	Sample location	Coordinates (N/E)	Altitude (m)	N	Haplotypes	h (S±D)	π (S±D) ×10^−3^
TSG	Tianshan group			**42**	5	0.718±0.042	10.393±5.243
BLK	Balikun, XJ	43°21'/93°42'	2085	14	H1,H2,H3,H4,H5	0.736±0.107	6.989±3.793
BY	Bayanbulak, XJ	42°84′/83°72′	2458	11	H1,H2,H3	0.618±0.104	10.575±5.757
HM	Hami, XJ	43°15′/93°38′	1921	8	H2	0	0
WLMQ	Urumqi, XJ	43°11′/86°85′	2031	9	H1,H2,H4,H5	0.750±0.112	7.053±4.015
QTPG	Qinghai-Tibetan Plateau group			**277**	17	0.889±0.011	7.344±3.690
SN1	Sunan, GS	39°02′/99°28′	2497	13	H1,H3,H5	0.641±0.097	3.161±1.845
SN2	Sunan, GS	38°3′/100°25′	2445	10	H4	0	0
TJ	Tianjun, QH	37°05′/98°52′	2533	16	H1,H2,H4,H5,H14	0.825±0.045	9.183±4.862
QL	Qilian, QH	38°05′/100°2′	2972	8	H1,H2,H3,H7	0.750±0.139	9.113±5.211
GC	Gangcha, QH	37°2′/100°31′	3428	12	H1,H2,H4,H5,H14	0.727±0.113	8.983±4.880
DT	Datong, QH	37°17′/101°24′	3712	14	H1,H2,H4,H7	0.692±0.094	4.282±2.408
HY	Huangyuan, GS	36°25′/101°13′	3375	10	H1,H4,H5,H7,H14	0.844±0.080	3.101±1.864
CD1	Chengduo, GS	33°21′/97°08′	4500	7	H1	0	0
XH1	Xunhua, QH	35°36′/102°41′	2792	8	H1,H3	0.536±0.123	3.017±1.876
SD	Shandan, GS	38°27′/101°11′	2358	15	H1,H3,H5	0.629±0.086	3.206±1.846
TZ	Tianzhu, GS	37°09′/102°5′	2613	11	H1,H2,H13	0.473±0.162	4.304±2.476
LT1	Lintan, GS	34°41′/103°34′	2800	7	H6,H7,H15	0.714±0.127	10.019±5.841
ZK1	Zeku, QH	35°18′/101°56′	2824	10	H1,H7,H14	0.689±0.104	2.033±1.293
ZK2	Zeku, QH	35°05′/101°36′	3426	10	H1	0	0
LX	Linxia, GS	35°34′/102°46′	3175	3	H1	0	0
GD	Gande, QH	34°00′/100°02′	4153	4	H1,H5,H7	0.833±0.222	1.566±1.261
LT	Litang, SC	30°1′/99°58′	3656	9	H1,H2,H4,H11,H12	0.833±0.098	7.795±4.413
KD	Kangding, SC	30°02′/101°3′	4346	8	H1,H11	0.536±0.123	0.336±0.352
DG	Dege, SC	31°41′/98°33′	3162	10	H1,H7,H9,H10,H17	0.867±0.071	5.705±3.246
DC	Daocheng, SC	29°07′/100°12′	3778	8	H6,H7	0.536±0.123	1.007±0.756
JD	Jiangda, XZ	31°32′/98°2′	3440	11	H2,H7,H10,H17	0.491±0.175	5.507±3.106
GZ	Ganzhi, SC	31°38′/99°48′	3385	6	H1,H10	0.333±0.215	0.418±0.425
YJ	Yajiang, SC	30°00′/100°4′	4173	7	H1,H2,H4,H9,H11	0.905±0.103	7.832±4.618
DZ	Dazhi, XZ	29°46′/91°50′	3910	10	H2,H7	0.467±0.132	8.766±4.866
MK	Mangkang, ZX	29°27′/98°38′	3681	6	H2,H8,H12	0.600±0.215	6.345±3.908
DQ	Dingqing, XZ	31°06′/96°21′	4321	10	H2,H6,H7,H8,H9	0.756±0.130	9.109±5.047
SD2	Songduo, XZ	29°52′/92°31′	4231	6	H1,H7	0,333±0.215	0.209±0.275
CD2	Changdu, XZ	31°21′/97°29′	3730	7	H1,H9,H16	0.667±0.160	11.211±6.508
SX	Suoxian, XZ	31°48′/93°43′	3979	12	H9,H12,H15,H17	0.455±0.170	4.523±2.567
NQ	Naqu, XZ	31°44′/92°39′	4432	9	H9,H12,H15,H16	0.778±0.110	10.604±5.919
**Total**				319	17	0.882±0.010	8.010±4.004

Abbreviation of Chinese Provinces: GS-Gansu, QH-Qinghai, SC-Sichuan, XJ-Xinjiang, XZ-Xizang (Tibet).

### DNA extraction, amplification and sequencing

Genomic DNA was extracted using a Plant Genomic DNA Isolation kit (Tiangen, Beijing, China) following the manufacturer’s instructions. The *trn*L-*trn*F [45] and *rpl*32-*trn*L [46] intergenic regions, widely used in plant phylogeographical analyses [21,30,32], were amplified and sequenced. PCR reactions were carried out in a total volume of 25 μL containing 20 ng template DNA, 2.5 μL PCR buffer, 2 μL MgCl_2_ (25 mmol/L), 0.5 μL dNTP mix (2.5 mmol/L), 1 μL each primer (5 pmol/L), and 0.3 μL (1 unit) Taq DNA polymerase. For DNA amplification a T1 thermo-cycler (Biometra, Göttingen, Germany) was used with an initial denaturation at 94°C for 3 min, followed by 32 cycles of denaturation at 94°C for 30 s, annealing at 53°C for 45 s, extension at 72°C for 1 min, and a final extension of 10 min at 72°C. The PCR products were checked on a 1.0% agarose gel, and then bidirectionally sequenced in a commercial laboratory (Sangon, Shanghai, China) following standard sequencing protocols.

### Genetic diversity and population structure

Chloroplast DNA (cpDNA) sequences were aligned with CLUSTAL W [47] and postprocessed manually. Insertions/deletions (indels) were coded as point mutations and received equal weight to other mutations. Chloroplast DNA haplotypes were identified based on variations in the aligned sequences of the *trn*L-*trn*F and *rpl*32-*trn*L spacers using DnaSP ver. 5.0 [48]. All of the cpDNA non-coding region of each chloroplast haplotype and outgroup were deposited in GenBank with the accession numbers KX180093-KX180130 (S1 Table). Haplotype diversity (*h*) and nucleotide diversity (π) for each population, for groups of populations and for all populations were calculated in ARLEQUIN 3.5 [49]. The effect of unequal sample sizes was assessed by rerunning analyses with alternative input files created through multiple random reductions [50] scripted in R ver. 3.2.3 [51]. No significant differences between the curtailed and the full data set were found so that we decided to proceed with analysis of the full dataset.

SAMOVA ver. 1.0 was used to investigate the spatial component in the dataset by defining *K* groups of populations that are geographically homogeneous and genetically differentiated from each other (10,000 iterations; range of 2 ≤ K ≤ 10) [52]. The result file, a pairwise cpDNA *F*_ST_ distance matrix, was imported into BARRIER [53] which incorporates Monmonier’s maximum-difference algorithm [54] to visualize the geographic location of genetic breaks among (groups of) populations. Using this method, we divided the distribution range of *P*. *kansuensis* populations into two groups, Tianshan group (TSG) and QTP group (QTPG). Furthermore, isolation by distance (IBD) [55], the correlation between genetic and geographical distance was checked with a Mantel test [56] using ALLELES IN SPACE (AIS) [57]. Genetic structure was assessed with an Analysis of Molecular Variance (AMOVA) [58] in ARLEQUIN 3.5 [49] with significance tests based on 10,000 permutations. Parameters of within-population gene diversity (*H*_S_), total gene diversity (*H*_T_), and genetic differentiation (*G*_ST_, *N*_ST_) were estimated according to Pons and Petit [59]. Significant phylogeographic structure was inferred by testing whether *N*_ST_ was significantly greater than *G*_ST_ using U-statistic. If *N*_ST_ is significantly higher than *G*_ST_, closely related haplotypes occur more often in the same populations than less closely related haplotypes, indicating the presence of phylogeographical structure [59].

### Phylogenetic relationship and divergence time

Phylogenetic relationships among *P*. *kansuensis* cpDNA haplotypes were analyzed using Neighbor-joining (NJ), Maximum parsimony (MP) and Maximum Likelihood (ML) algorithms implemented in MEGA ver. 6.0 [60], with *P*. *violascens* and *P*. *verticillata* as outgroups. Gaps in sequences were treated as the fifth character state. We constructed MP trees using a heuristic search with 1,000 random additions of sequences and tree-bisection reconnection (TBR) branch swapping. The ML and MP trees were computed with 1,000 bootstrap replicates in Kimura’s two-parameter model. Furthermore, NETWORK ver. 4.6 [61] was used to construct median-joining networks to detect genealogical relationships among the haplotypes of *P*. *kansuensis*. The gaps were treated as a single mutation event.

Divergence times for different *P*. *kansuensis* lineages were estimated through a Bayesian approach implemented in Beast ver. 1.8.1 [62]. In running MODELTEST ver. 3.7 [63], generalized time reversible (GTR) substitution model and Gamma site heterogeneity model were selected as the best-fit nucleotide substitution model for our dataset of aligned sequences. Due to a lack of fossils of *P*. *kansuensis* or its congeneric relatives, substitution rates were used for approximate divergence times. For most angiosperms, the cpDNA substitution rates are estimated to vary between 1.0 and 3.0×10^−9^ substitutions per site per year [s/s/y], while 8.24×10^−9^ for *trn*L-*trn*F [64]. Because *P*. *kansuensis* is an annual or biennial herb and *trn*L-*trn*F was used in this study, the value of 3.0 and 8.24×10^−9^ was specified in BEAST with an additional uncorrelated lognormal relaxed molecular clock assumption. The Markov chain Monte Carlo (MCMC) chains were run for 10,000,000 generations, sampling every 1,000 generations. The combined parameters were checked in TRACER ver. 1.5 [65]. The Bayesian trees were combined and annotated by TREE ANNOTATOR ver. 1.8.1 (part of the BEAST 1.8.1 package).

### Population demographic analyses

To investigate whether populations or groups of populations experienced any population expansion, Tajima’s *D* [66] and Fu & Li’s D* [67] were calculated using ARLEQUIN 3.5 [49]. In addition, mismatch distribution analysis was also calculated in ARLEQUIN with 1,000 parametric bootstrap replicates. The sum of squared deviations (SSDs) between observed and expected mismatch distribution were computed and *P* values were calculated as the proportion of simulations producing a larger SSD than the observed SSD. The raggedness index (*HRag*) and its significance were also calculated to quantify the smoothness of the observed mismatch distribution [68].

### Species distribution modelling

Lastly, in order to estimate the current potential distribution range of *P*. *kansuensis* as well as during the Last Glacial Maximum (LGM; 21 ka before present), a species distribution model (SDM) was computed using the maximum entropy algorithm implemented in MAXENT 3.3.1 [69]. Present day climate data available from the World Clim database (34 stations, 19 bioclimatic variables, 2.5 arcmin resolution) [70] (available at http://www.worldclim.org/download) along with 34 tested geographical data produced by ourselves were used to estimate the present potential distribution range. The community climate system model (CCSM) [71] was then employed to generate the potential distribution during the LGM. To test the reliability of the results, goodness of fit between the model and the training data was assessed by analyzing the area under the receiver operating characteristic curve (AUC). Finally, a jackknife test was performed to measure the relative importance of climatic variables on the occurrence prediction for every distribution model.

## Results

### Chloroplast variation and haplotype distribution

**Fig 1:**
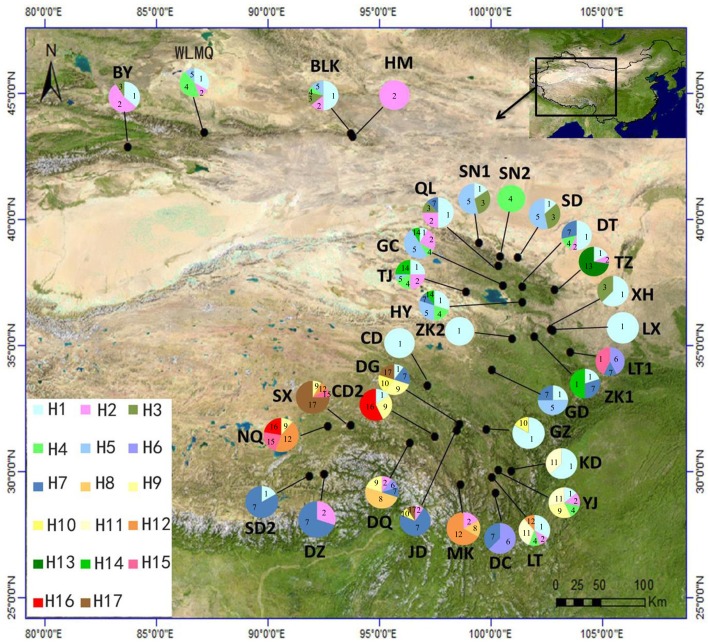
Sample sites (population codes as in Table 1) and geographic distribution of chloroplast DNA (cpDNA) haplotypes (H1-H17) detected in 34 populations of *Pedicularis kansuensis* in the Qinghai-Tibetan Plateau (QTP) and the Tianshan region (TSR). Pie charts show the different haplotypes and their frequency in each population.

The aligned sequences of *trn*L-*trn*F and *rpl*32-*trn*L were 830 bp and 770 bp in length, respectively, with a total length of the combined alignments of 1,600 bp. Variable sites showed 40 substitutions and 15 indels. In total, 17 haplotypes (H1-H17) were identified (Fig 1, Table 1). Among these, H1 and H2 were widespread haplotypes, occurring in 23 (67.65%) and 16 (47.06%) populations, respectively. All 17 haplotypes were found in the QTP and only five in the TSR (H1-H5). Thus, no haplotype was exclusive for the TSR. Population contained a maximum of five haplotypes and a minimum of one. There was no significant correlation between the number of sampled individuals per population and the number of haplotypes (*R* = 0.3; P > 0.05).

### Genetic diversity and structure

Haplotype diversity (*h*) ranged from 0.000 to 0.905 and the YJ (Yajiang) population in the Sichuan Hengduan Mts. contributed the highest value (Table 1, Fig 1). Nucleotide diversity (π) varied between 0.000 and 11.210×10^−3^ with a maximum present in the CD2 (Changdu) population in SE Tibet (Table 1, Fig 1). Total genetic diversity based on haplotype variation across all populations was *H*_T_ = 0.882 and the average within-population diversity was *H*_S_ = 0.559 (Table 2).

**Table 2:** Estimates of average gene diversity results for *Pedicularis kansuensis* within regions.

Region	*H*_S_	*H*_T_	*G*_ST_	*N*_ST_
**All data**	0.559 ±0.047	0.882 ±0.025	0.366 ±0.050	0.376 ±0.069
**Tianshan group**	0.526 ±0.178	0.753 ±0.109	0.301 ±0.225	0.481 ±0.269
**QTP group**	0.556 ±0.051	0.880 ±0.029	0.368 ±0.051	0.386 ±0.074

Abbreviations: *H*_S_—average gene diversity within populations; *H*_T_—total gene diversity; *G*_ST_—inter population differentiation; *N*_ST_—number of substitution types.

**Fig 2:**
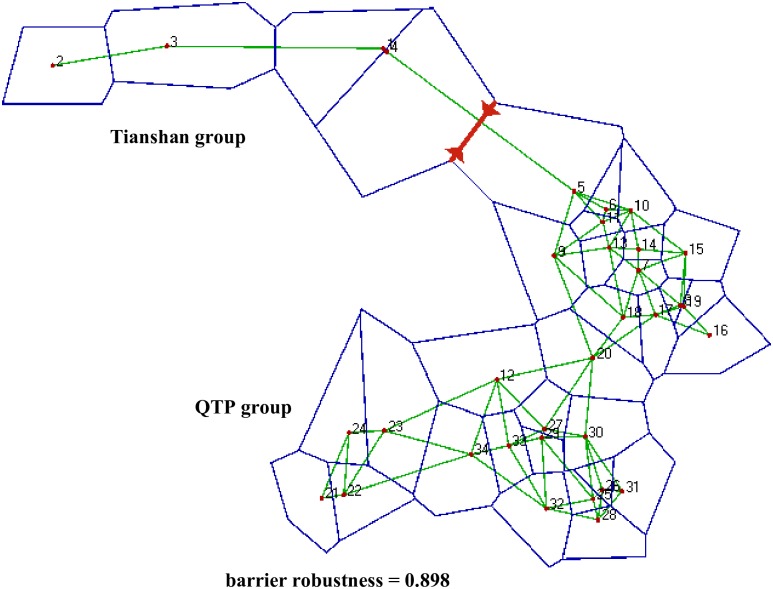
Location of inter-population genetic breaks in *Pedicularis kansuensis* in the Qinghai-Tibetan Plateau (QTP) and Tianshan region (TSR). Outlines represent the polygons of the Voronoï tessellation with the centers of the populations omitted. Red line represent the barrier. The distribution range of *P*. *kansuensis* was divided into two groups, Tianshan group (TSG) and QTP group (QTPG).

The permutation test showed that there was no significant difference between *G*_ST_ = 0.366 and *N*_ST_ = 0.376 (*U* = 0.11; P > 0.05). Thus, the hypothesis of a strong phylogeographic pattern was rejected. In the SAMOVA analyses, *F*_CT_ values decreased progressively as the values for *K* number of groups increased from 2 to 10 with no unambigous number of *K* supported. Also here the hypothesis of a phylogeographic pattern was rejected. Furthermore, the Mantel test revealed a significant correlation between genetic and geographical distances (*R* = 0.127, P < 0.001) over all populations. However, a genetic break (barrier) separating the TSR populations from those of the QTP was found with a robustness of 90% (Fig 2). This barrier corresponds to the arid land between the two disjunctive geographic regions. Hierarchical analysis of molecular variance (AMOVA) showed that a low variation (2.52%) was partitioned to the two putative groups of populations, while 33.03% and 64.44% variation was partitioned among populations within groups and within populations, respectively (Table 3).

**Table 3:** Analysis of molecular variance for 34 sampled populations of *Pedicularis kansuensis* based on two cpDNA spacer sequence data.

Source of variation	d.f.	Sum of squares	Variance components	Percentage of variation	Fixation Index
**Among groups**	1	2.763	0.01146	2.52	*F*_SC_: 0.33886
**Among pops. within groups**	32	54.029	0.14998	33.03	*F*_ST_: 0.3555
**Within pops.**	285	83.397	0.29262	64.44	*F*_CT_: 0.02525
**Total**	318	140.188	0.45895		

Abbreviation: d.f.—degrees of freedom. *F*_CT_—correlation of chlorotypes within groups relative to the total; *F*_SC_—correlation within populations relative to groups; *F*_ST_—correlation within populations relative to the total.

### Phylogenetic and genealogical relationships of cpDNA haplotypes

**Fig 3:**
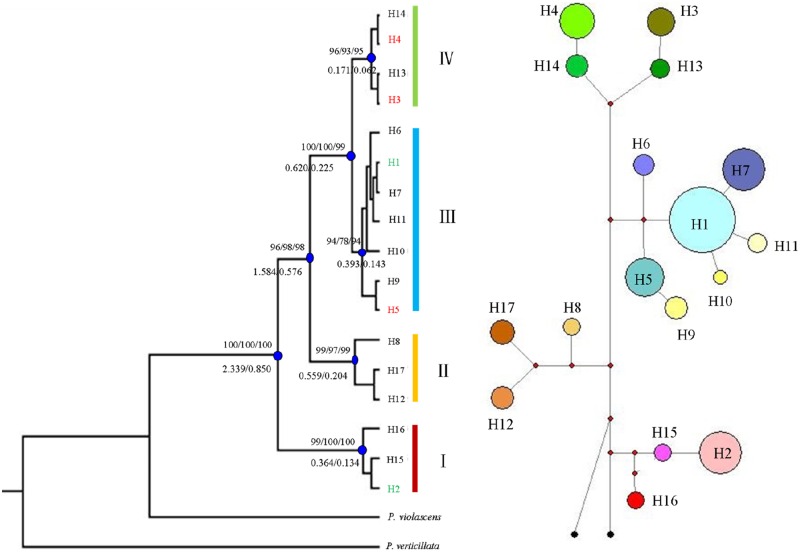
The NJ tree topology (left) and network (right) of the 17 cpDNA haplotypes detected in *Pedicularis kansuensis* and their divergence times estimated with the average evolutionary rate based on BEAST analysis. The values above the branch represent the bootstrap values for NJ (left), MP (middle) and ML (right) analyses, respectively. The values under the branching represent the divergence time (in million years ago), based on 3.0×10^−9^ substitutions per site per year [s/s/y] and 8.24×10^−9^ s/s/y. The circle sizes in the network are proportional to haplotype frequency, and the black points represent outgroups.

The topology of the Neighbor-joining (NJ) tree calculated for 17 haplotypes from 319 *P*. *kansuensis* individuals is shown in Fig 3. Four clades were strongly supported (≥ 94% bootstrap support). The haplotypes in clade I and II mainly occurred in populations from the SE of the QTP with the exception of H2, a widespread haplotype present in 16 populations. Clade IV contained four haplotypes which all stem from the NE edge of the QTP and the TSR. Clade III was the most complicated one, containing 7 haplotypes distributed in 33 populations. Among these haplotypes, H1 represented the most widespread haplotype in our study, occurring in 23 populations. H9-H11 were found in the SE of the QTP. H5 was found in the NE of the QTP and in the TSR. The results of the median-joining network obtained by NETWORK ver. 4.6 [61] showed the same phylogenetic relationship as those revealed by the NJ tree (Fig 3). Also, the maximum parsimony (MP) and maximum likelihood (ML) trees were essentially identical to the NJ tree with respect to the major clades and were thus not shown here.

### Lineage divergence time and population spatial expansion

**Fig 4:**
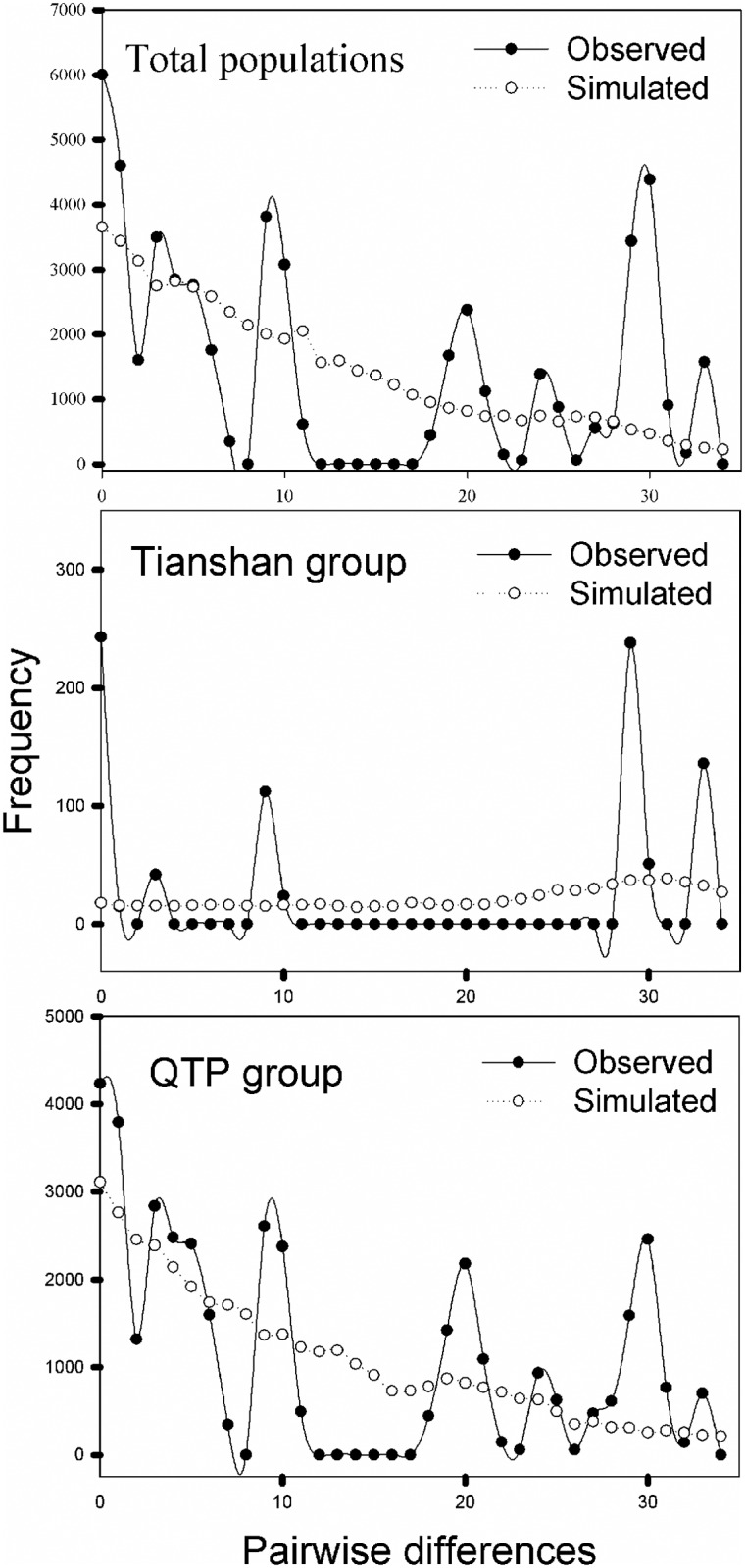
Mismatch distribution analysis of cpDNA sequence data from all sampled populations in the Qinghai-Tibetan Plateau group (QTPG) and the Tianshan group (TSG).

Divergence times between the haplotypes ranged from 2.339 to 0.034 Mya when the value of 3.0×10^−9^ s/s/y was specified in BEAST, while 0.885 to 0.012 Mya for 8.24×10^−9^ s/s/y (Fig 3). The mismatch distribution for pairwise differences over all populations and two geographical groups were clearly multimodal (Fig 4), indicating that this species has not experienced a sudden expansion. This was corroborated by positive and insignificant Tajima’s *D* and Fu & Li’s D* tests (Table 4).

**Table 4:** Parameters of mismatch distribution analyses, Tajima’s D and Fu & Li’s D* tests.

Groups	Mismatch distribution analyses		Tajima’s D test (*P*)	Fu & Li’s D* tests (*P*)
	SSD (*P*_*SSD*_)	HRag (*P*_*HRag*_)		
**Total**	0.024(0.500)	0.028(0.120)	1.310(0.906)	16.720(0.982)
**Tianshan group**	0.096(0.120)	0.280(0.390)	3.126(0.999)	21.651(1.000)
**QTP group**	0.015(0.640)	0.022(0.510)	0.856(0.814)	13.363(0.999)

Abbreviations: *HRag* (*P*_*HRag*_)—raggedness statistic (probability of raggedness statistic); *SSD* (*P*_*SSD*_)—sum of the square deviations (probability of sum of the square deviations).

### Species distribution modeling

**Fig 5:**
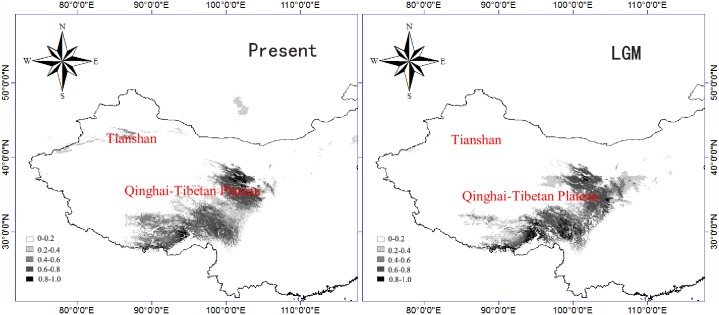
Potential distribution range for *Pedicularis kansuensis* under current climate conditions (left) and during the Last Glacial Maximum (LGM; right) based on the community climate system model (CCSM).

The 'area under the curve' (AUC) values for the training and the test data of *P*. *kansuensis* amounted to 0.998 and 0.995, respectively, indicating good performance of the present-day distribution range and the Last Glacial Maximum community climate system model (LGM-CCSM) as visualized in Fig 5. The current potential distribution range of *P*. *kansuensis* included the east of the QTP as well as the TSR, coinciding well with the species’ extant distribution. By contrast, the predicted distribution for the LGM showed occurrences only in the east of the QTP.

## Discussion

### Genetic Diversity and Genetic Structure

In this study, we detected 17 haplotypes from 319 *P*. *kansuensis* individuals belonging to 34 populations in the QTP and the TSR. In comparison with *P*. *longiflora*, a congeneric species of *P*. *kansuensis* which shares many life-history traits (e.g. annual/biennial, insect-pollinated, outcrossing, mid-successional) and has an almost matching distribution range on the QTP with northernmost occurrences in either the Tianshan or the Altay Mts. [43,72,73], the haplotype diversity was slightly higher in *P*. *kansuensis* (*H*_T_ = 0.882 *vs*. *H*_T_ = 0.770), though the number of haplotypes found was less than that in *P*. *longiflora* (30 haplotypes, 41 populations, 910 individuals) [35]. When compared with less related plant species studied in both the QTP and the Tianshan region, haplotype diversity of *P*. *kansuensis* was also higher (e.g. *Aconitum gymnandrum* (*H*_T_ = 0.739) [74], *Angelica nitida* (*H*_T_ = 0.818) [75], *Cupressus* spp. (*H*_T_ = 0.249 to *H*_T_ = 0.791) [76], *H*. *vulgaris* (*H*_T_ = 0.604) [37], *Juniperus sabina* (*H*_T_ = 0.57) [21] and the Chinese populations of *Ligularia hodgsonii* (*H*_T_ = 0.869) [77]). This comparison still holds true when only haplotype diversity of populations from the TSR are considered (Tables 1 and 2) [35–37], despite the fact that *P*. *kansuensis* haplotype diversity was lower in the TSR than in the QTP (*H*_T_ = 0.753 *vs*. *H*_T_ = 0.880). Unlikely, the high level of total genetic diversity can be attributed to a short life-history trait such as longevity or the outcrossing breeding system of *P*. *kansuensis*. Considerable gene flow among populations and regions may have played a role in shaping the genetic structure, as deduced from the low levels of genetic differentiation among populations in the entire study area (*G*_ST_ = 0.366), the QTP (*G*_ST_ = 0.368), or the TSR (*G*_ST_ = 0.301) (Table 2).

Gene flow of cpDNA is only possible via means of seeds or clonal plant fragments. [78,79]. In the absence of asexual means for reproduction in *P*. *kansuensis*, our results, i.e. high genetic diversity within population and low population differentiation, suggest relatively frequent seed exchange among populations. The seeds of *P*. *kansuensis*, however, have no obvious morphological adaptations to wind, water or animal dispersal [80,81]. Nevertheless, water flow has been shown to be an effective way of seed dispersal for *P*. *kansuensis* [82], but the hydrography of the Tarim Basin makes this option improbable. Also, secondary wind dispersal across frozen land surfaces seems unlikely given the elevational gradients and northerly winter wind direction [83]. Animal activities, especially migratory birds, as well as transportation of contaminated herbage seeds may have played a role in the dispersal of seeds across the Tarim Basin [84–86] despite the fact that direct observations are lacking. These latter two options could well explain why we did not find a strong phylogeographic signal with a clear separation of populations from the QTP and the TSR. The SAMOVA results rendered no support for a distinct number of *K* groups of populations, the comparison of *G*_ST_ and *N*_ST_ values showed no significant difference (Table 2; *U* = 0.11; P > 0.05) and only 2.52% of the molecular variance could be contributed to differences among the two regions (Table 3). The genetic barrier is thus very weak despite being detected with high robustness (Fig 2) in BARRIER. Just like the limited available studies of species with a similar distribution pattern, we found no convincing evidence for genetic differentiation in *P*. *kansuensis* between the QTP and the TSR [35–37].

### Extensive survival in the QTP through the Quaternary

The divergence of all *P*. *kansuensis* haplotypes could be dated back to 2.339 (0.850) Mya time window that coincides with the early or middle Pleistocene, suggesting that *P*. *kansuensis* withstood the extensive climate changes during the Quaternary. During this period, the QTP had experienced four major glaciations [87] and several glacial and interglacial cycles [88]. Based on the estimated divergence times of main lineages and most of the haplotypes, we presume that the Quaternary climatic oscillations may have greatly shifted distribution range of *P*. *kansuensis* in the QTP, affected its divergence events, and shaped its phylogeographic structure, just as reported in other plant species [1–4,28,29,89]. Based on recent phylogeographical studies in the demographic history of plant species from the QTP, two main refugium hypotheses have been proposed. One hypothesis suggested some species may have retreated to the eastern or south-eastern plateau edge (e.g. Hengduan Mts.) as refugia during the Quaternary glacial periods, and then recolonized QTP and its surrounding regions during the interglacial phases or at the end of the Last Glacial Maximum (LGM) [35,90–95]. While the other hypothesis suggested some species may have also survived at QTP and its surrounding regions *in situ* through the Quaternary [96,97]. Previous studies in NW China showed species survival in East Tianshan Mountains [22] and Ili (Yili) Valley [33] during the Quaternary. Refugia are usually correlated with high levels of genetic diversity and unique haplotypes [98]. In this study, H1 and H2 were widespread haplotypes, occurred in 23 (67.65%) and 16 (47.06%) populations, respectively. Some haplotypes (e.g. H8, H9, H10, H11, H15, H16, and H17) occurred in the SE QTP, Hengduan-Himalayan Mts. While H3, H5, H13, and H14 occurred in the NE QTP (e.g. Qilian Mts.) and Tianshan Mts. It seems that *P*. *kansuensis* might have survived in known refugial areas at SE QTP (e.g. Hengduan Mts.) and the edge of NE QTP (e.g. Qilian Mts.). However, the results of mismatch analyses (Fig 3) and Tajima’s D and Fu & Li’s D* tests (Table 3) indicated that recent range expansion was rejected, given that *P*. *kansuensis* survived extensively in the plateau during the LGM. This was also confirmed by the results of SDM that the LGM potential distributions did not show obviously shrink in the QTP in comparison with the current distributions (Fig 5).

### Long-distance dispersal from the QTP to the TSR after the LGM

In this study, all 17 detected haplotypes were found in the QTP, while only five (H1-H5) in the TSR (Fig 1). All five haplotypes (H1-H5) found in the TSR also occurred in NE of the QTP, of which H1 and H2 were widespread over the entire distribution range (Fig 1). In the phylogenetic tree, neither the five (H1-H5) nor the three (H3-H5) haplotypes formed a single clade, but rather clustered with other haplotypes (Fig 3). The divergence time of H1-H5 corresponded to the divergence time of all haplotypes and was dated back to the early Pleistocene, at 2.339 (0.850) Mya, while the one for H3-H5 to 0.620 (0.225) Mya, and H3 and H4 to 0.17 (0.062) Mya. Furthermore, a wide arid region barrier between the TSR and the QTP had developed and aridification begun by the early Pleistocene [99]. The divergence times of the shared haplotypes were later than the enlarging of aridification (Fig 3). The predication was confirmed by the low molecular variance between groups (2.52%, Table 3). Therefore, the disjunctive distribution of *P*. *kansuensis* was unlikely the result of a range fragmentation, but shaped by long-distance dispersal crossing the wide arid land. Generally, long-distance dispersal is characterized by a movement from high genetic diversity region to low genetic diversity region [23,25]. The index of genetic diversity (*H*_T_) of the QTPG is significant higher than the TSG (*H*_T_ = 0.880 *vs*. *H*_T_ = 0.753) (Table 2). By this token, long-distance dispersal throughout arid land from the QTP, especially the northeast of the QTP, to the TSR could be the reason for the disjunctive distribution of *P*. *kansuensis*. In *P*. *longiflora*, the single haplotype that genetically connected the Altay Mts. with the NE of the QTP was also estimated to have diverged around 0.138 Mya. Both *P*. *longiflora* and *P*. *kansuensis* show lower level of genetic diversity in the Tianshan-Altay region than of the QTP. Given that the cradle of the genus *Pedicularis* is likely in the Hengduan-Himalayan Mts. at the SE of the QTP [100], the NW Chinese Tianshan and Altay Mts. were presumably colonized from the QTP earliest during the last interglacial of the late Pleistocene [87]. At that time the arid land barrier between the different mountain ranges as seen today must have been discontinuous to allow for seed flow. Evidence for this scenario is however lacking.

The species distribution model (SDM) results show an absence of *P*. *kansuensis* from the TSR during the LGM (Fig 5). This indicates that colonization must have occured after the LGM, hence rather recently. This corroborates the genetic findings. Nevertheless, the reliablity of the SDM is to be taken with caution as we failed to detect any range expansion or contraction in the QTP which would be an intuitive assumption (Table 3, Fig 3).

## Conclusion

Based on phylogeographical and species distribution modeling analyses, we propose that *P*. *kansuensis* has survived on the QTP throughout the LGM. The present day disjunct distribution in the Qinghai-Tibetan Plateau and the Tianshan Region is likely the result of multiple bird or human assisted long-distance seed dispersal events crossing the arid land of Tarim Basin after the LGM, particularly from the northeastern fringes of the QTP to the Tianshan Mts.

## Supporting Information

S1 Table The detailed voucher specimens information for used samples, including outgroup, and the GenBank accession numbers of the used sequences. (DOCX)
