# Supplementary material for: Long-Distance Dispersal after the Last Glacial Maximum (LGM) Led to the Disjunctive Distribution of Pedicularis kansuensis (Orobanchaceae) between the Qinghai-Tibetan Plateau and Tianshan Region
Source: PLoS One. 2016 Nov 2;11(11):e0165700. doi: 10.1371/journal.pone.0165700 (PMC5091882; doi:10.1371/journal.pone.0165700)
Supplement: S1 Table — (DOCX) [file pone.0165700.s001.docx]

Table S1. The detailed voucher specimens information for used samples, including outgroup, and the GenBank accession numbers of the used sequences.

| Species | Haplotypes | Voucher specimens (population) | Accession numbers | |
| --- | --- | --- | --- | --- |
|  |  |  | *rpl*32-*trn*L | *trn*L-*trn*F |
| *Pedicularis kansuensis* | H1 | Li WJ Liu YY Lu J 0832 (BY);  Li WJ Liu YY Lu J 0861(WLMQ);  Li WJ Liu YY Lu J 0002(BLK);  Li AR Sui XL GZ05(GZ);  Li AR Sui XL KD05(KD);  Li AR Sui XL LT02(LT);  Li AR Sui XL YJ08(YJ);  Li AR Sui XL CD05(CD2);  Li AR Sui XL DG07(DG);  Li AR Sui XL SD13(SD2);  Li WJ Liu YY Lu J 0094(SN1);  Li WJ Liu YY Lu J 0170(TZ);  Li WJ Liu YY Lu J 0721(DT);  Li WJ Liu YY Lu J 0788(GC);  Li WJ Liu YY Lu J 0806(TJ);  Li WJ Liu YY Lu J 0156(SD);  Li WJ Liu YY Lu J 0626(CD);  Li WJ Liu YY Lu J 0529(GD);  Li WJ Liu YY Lu J 0697(HY);  Li WJ Liu YY Lu J 0258(LX);  Li WJ Liu YY Lu J 0304(XH);  Li WJ Liu YY Lu J 0339(ZK1);  Li WJ Liu YY Lu J 0742(QL); | KX180093 | KX180112 |
|  | H2 | Li WJ Liu YY Lu J 0839(BY);  Li WJ Liu YY Lu J 0871(WLMQ);  Li WJ Liu YY Lu J 0011(BLK);  Li AR Sui XL LTF01(LT);  Li AR Sui XL DQ08(DQ);  Li AR Sui XL YJFB03(YJ);  Li WJ Liu YY Lu J 0173(TZ);  Li WJ Liu YY Lu J 0724(DT);  Li WJ Liu YY Lu J 0772(GC);  Li WJ Liu YY Lu J 0058(HM)  Li WJ Liu YY Lu J 0807(TJ);  Li WJ Liu YY Lu J 0748(QL);  Li WJ Liu YY Lu J 0058(SN1);  Li AR Sui XL DZ05(DZ);  Li AR Sui XL JD11(JD);  Li AR Sui XL MK03(MK); | KX180094 | KX180113 |
|  | H3 | Li WJ Liu YY Lu J 0834(BY);  Li WJ Liu YY Lu J 007(BLK);  Li WJ Liu YY Lu J 0078(SN1);  Li WJ Liu YY Lu J 0152(SD);  Li WJ Liu YY Lu J 0303(XH);  Li WJ Liu YY Lu J 0743(QL); | KX180095 | KX180114 |
|  | H4 | Li WJ Liu YY Lu J 0864(WLMQ);  Li WJ Liu YY Lu J 0013(BLK);  Li AR Sui XL LT04(LT);  Li AR Sui XL YJ09(YJ);  Li WJ Liu YY Lu J 0737(DT);  Li WJ Liu YY Lu J 0789(GC);  Li WJ Liu YY Lu J 0800(TJ);  Li WJ Liu YY Lu J 0690(HY);  Li WJ Liu YY Lu J 0102(SN2) | KX180096 | KX180115 |
|  | H5 | Li WJ Liu YY Lu J 0883(WLMQ);  Li WJ Liu YY Lu J 0003(BLK);  Li WJ Liu YY Lu J 0075(SN1);  Li WJ Liu YY Lu J 0786(GC);  Li WJ Liu YY Lu J 0808(TJ);  Li WJ Liu YY Lu J 0148(SD);  Li WJ Liu YY Lu J 0527(GD);  Li WJ Liu YY Lu J 0691(HY); | KX180097 | KX180116 |
|  | H6 | Li AR Sui XL DC03(DC);  Li AR Sui XL DQ01(DQ);  Li WJ Liu YY Lu J 0236(LT1); | KX180098 | KX180117 |
|  | H7 | Li AR Sui XL DC06(DC);  Li AR Sui XL DQ09(DQ);  Li WJ Liu YY Lu J 0729(DT);  Li WJ Liu YY Lu J 0237(LT1);  Li AR Sui XL DZ01(DZ);  Li AR Sui XL DG08(DG);  Li AR Sui XL JD02(JD);  Li AR Sui XL SD05(SD2);  Li WJ Liu YY Lu J 0525(GD);  Li WJ Liu YY Lu J 0693(HY);  Li WJ Liu YY Lu J 0341(ZK1);  Li WJ Liu YY Lu J 0739(QL); | KX180099 | KX180118 |
|  | H8 | Li AR Sui XL DQ03(DQ);  Li AR Sui XL MK01(MK); | KX180100 | KX180119 |
|  | H9 | Li AR Sui XL DQ06(DQ);  Li AR Sui XL YJ01(YJ);  Li AR Sui XL CD06(CD2);  Li AR Sui XL DG04(DG);  Li AR Sui XL NQ02(NQ);  Li AR Sui XL SX06(SX); | KX180101 | KX180120 |
|  | H10 | Li AR Sui XL GZ12(GZ);  Li AR Sui XL DG02(DG);  Li AR Sui XL JD01(JD); | KX180102 | KX180121 |
|  | H11 | Li AR Sui XL KDF02(KD);  Li AR Sui XL LT03(LT);  Li AR Sui XL YJ03()YJ; | KX180103 | KX180122 |
|  | H12 | Li AR Sui XL LT05(LT);  Li AR Sui XL MK06(MK);  Li AR Sui XL NQ04(NQ);  Li AR Sui XL SX09(SX); | KX180104 | KX180123 |
|  | H13 | Li WJ Liu YY Lu J 0168(TZ); | KX180105 | KX180124 |
|  | H14 | Li WJ Liu YY Lu J 0774(GC);  Li WJ Liu YY Lu J 0805(TJ);  Li WJ Liu YY Lu J 0700(HY);  Li WJ Liu YY Lu J 0338(ZK1); | KX180106 | KX180125 |
|  | H15 | Li WJ Liu YY Lu J 0238(LT1);  Li AR Sui XL NQF01(NQ);  Li AR Sui XL SX07(SX); | KX180107 | KX180126 |
|  | H16 | Li AR Sui XL CD03(CD2);  Li AR Sui XL NQ03(NQ); | KX180108 | KX180127 |
|  | H17 | Li AR Sui XL DG03(DG);  Li AR Sui XL JD09(JD);  Li AR Sui XL SX01(SX); | KX180109 | KX180128 |
| *P. violascens* | H18 | Li WJ 13039 | KX180110 | KX180129 |
| *P. verticillata* | H19 | Li WJ Liu YY Lu J 0133 | KX180111 | KX180130 |
